# A Guttate Psoriasis That Tends to Spare Three Tattoos: A Macrophage Liaison

**DOI:** 10.1155/2021/9448636

**Published:** 2021-09-11

**Authors:** Panagiota Spyridonos, Vasiliki Zampeli, Sophia-Nefeli Rapti, Ioannis D. Bassukas

**Affiliations:** ^1^Department of Medical Physics/Informatics, Faculty of Medicine, School of Health Sciences, University of Ioannina, Ioannina, Greece; ^2^Department of Skin and Venereal Diseases, Faculty of Medicine, School of Health Sciences, University of Ioannina, Ioannina, Greece; ^3^Department of Dermatology, University Hospital of Ioannina, Ioannina, Greece

## Abstract

Induction of new psoriasis sites was reported in only a small amount of psoriasis patients undergoing tattooing, despite the intuitive belief that tattoo trauma might awaken the disease due to the isomorphic phenomenon of Koebner. In this case report, we discuss a patient who presented with a remarkable sparing of his three tattoo sites during a guttate psoriasis flare-up that was unrelated to tattooing. The spatial concordance of tattoo and psoriasis lesions was analyzed on clinical pictures of tattoo sites taken during the psoriasis episode. For the quantification of the spatial distribution of the psoriasis lesions, Voronoi diagrams were generated, and coefficients of variation and the two-sample *t*-test were employed to compare the distributions of Voronoi patch sizes in different settings. Compared to skin areas without tattoos, a tattoo introduced a higher variation in the sizes of the Voronoi patches centered around psoriasis lesions. Based on our findings, we would like to discuss the possible role of macrophages as the key cellular link in the complex pathophysiologic relationship between tattooing/tattoo and psoriasis. Taking into account the relationship of autophagy and psoriasis lesions, we propose the hypothesis that tattoos represent a “psoriasis-hostile” tissue environment pertained by a population of LAP active M2-polarized macrophages. Further clinical studies of the relationship of psoriasis lesions to the tattooed skin are needed and may provide important insights into the role of macrophages in the pathogenesis of psoriasis.

## 1. Introduction

The nosologic relationship of psoriasis and tattoo remains a matter of debate. Despite the intuitive concern that tattoo trauma might induce the disease (Koebner phenomenon, KP), a KP was reported in only 3.2% of psoriasis patients undergoing tattooing [1], an overall rather moderate rate [2]. Moreover, Grodner and colleagues [3] observed that much more patients with psoriasis did not recall lesions that had affected their tattoo sites during episodes of disease flare-ups (47.6%) than recalling them (19.8%). Herein, on the occasion of a patient who presented with a distinct sparing of three tattoo sites during a guttate psoriasis episode that was unrelated to tattooing, we would like to comment on the putative role of macrophages as the key cellular link in the complex pathophysiologic relationship between tattooing/tattoo and psoriasis.

## 2. Case Presentation

**Figure 1:**
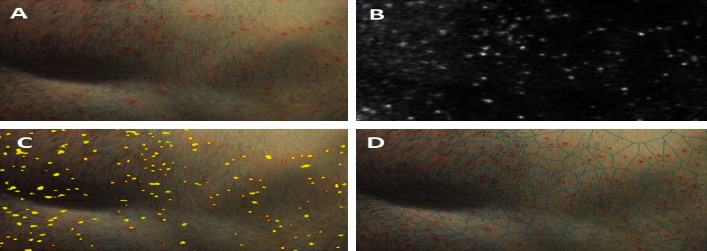
(a) Skin area affected with psoriasis. (b) Enhanced lesion representation by a^*∗*^ color channel of lab space. (c) Segmented lesions (yellow spots) superimposed on the initial image to visualize the result of morphological processing. (d) Lesions' centroids generating Voronoi tessellation.

**Figure 2:**
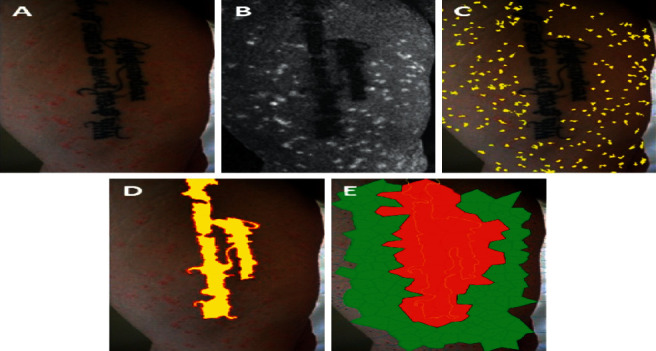
Psoriasis and tattoo #1. (a) Clinical picture. (b) Retained a^*∗*^ color channel of L^*∗*^a^*∗*^b^*∗*^ color space: high-peak intensity areas mark psoriasis lesions and low-level intensity areas correspond to tattooed skin segments. (c) Applying h-maxima transform followed by regional maxima, the lesional skin spots (patches) were extracted and are displayed in yellow pseudocolor. (d) Equivalently, the tattoo area was extracted by applying h-minima transform, followed by regional minima and is shown in yellow pseudocolor to enhance visualization. (e) The extracted psoriasis skin lesions' centroids (panel (C) applied to generate Voronoi diagrams (tessellation) of the tattoo area. Voronoi polygons with and without overlapping with the tattoo area (yellow border) are displayed in red and green pseudocolors, respectively.

**Figure 3:**
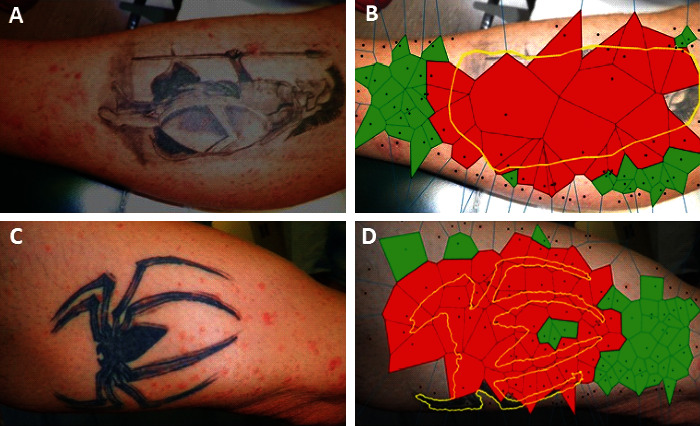
Tattoo #2 (panels (a) and (b)) and tattoo #3 (panels (c) and (d)). (a)–(c) Clinical pictures. (b)–(d) Voronoi patches intersecting (red) or not intersecting (green) tattooed skin areas (yellow contoured).

An afebrile, 27-year-old male patient, in otherwise good general condition, presented with a rash of scattered, up to 1.0 cm large psoriasiform lesions that progressively affected his torso (shown in Figure 1) and the extremities during the past two weeks. The rash was accompanied by moderate itching and an apparent tendency to coalesce in typical for plaque psoriasis skin localizations, i.e., the knees. The patient was diagnosed with plaque psoriasis at the age of 15 years, which was treated successfully with topical regimens. The laboratory evaluation including chest X-ray, ASO, syphilis, and hepatitis A, B, and C serology returned no pathologic findings. Based on patient's history, the macromorphology of the rash, and a lesional skin biopsy, guttate psoriasis was diagnosed. Except for psychosocial stress, no other triggering events of the dermatosis could be elucidated. Remarkably, the rash spared almost completely the sites of his three tattoos (lower leg, forearm, and shoulder; larger diameters 15–25 cm, shown in Figures 2 and 3). The tattoos (two of them monochrome black and the third black contours on whitish background; Figures 2 and 3) were performed on different occasions over the last 3 years (the last one about 9 months before the flare-up) in the same tattoo studio. The patient reported no KP on previous tattooing with either local or generalized exacerbations of his psoriasis.

The spatial concordance of tattoo and psoriasis lesions was analyzed on clinical pictures of tattoo sites taken during the flare-up. For the detection of psoriasis lesions, RGB clinical images (shown in Figure 1(a)) were converted into the L^*∗*^a^*∗*^b^*∗*^ color space. The high-peak intensity areas (“spots”) in the a^*∗*^color channel (shown in Figure 1(b)), which correspond to psoriasis lesions, were extracted by serially applying the h-maxima transform and the regional maxima morphological operations [4] (shown in Figure 1(c)). To quantify the spatial distribution of the psoriasis lesions, Voronoi diagrams [5] were generated with generator points the centroids of above intensity spots (shown in Figure 1(d)).  Figure 2 exemplifies this procedure in the case of a tattoo (arbitrary designated tattoo #1). Coefficients of variation (CV) and two-sample *t*-test were employed to compare the distributions of Voronoi patch sizes in different settings. Skin areas without a tattoo, like the front of the torso (Figure 1), are characterized by a rather homogeneous, unimodal size distribution of the Voronoi patches centered on psoriasis lesions (CV = 0.46, Table 1). However, the presence of a tattoo introduces a higher variation in the size distributions of the corresponding Voronoi patches in its neighborhood (CV = 0.60–1.23; shown in Figures 2 and 3; Table 1). Moreover, it seems that the presence of a tattoo induces a bimodal size distribution of the Voronoi polygons, with the patches that intersect tattoo lines, i.e., those patches that are localized within the tattooed skin area or are located in the vicinity of the tattoo contours, being significantly larger compared to the rest, more remote polygons (*p* < 0.001; Table 1).

## 3. Discussion/Conclusion

Sparing of skin lesions by an evolving psoriasis has been observed, so psoriasis spared a polio-affected limb [6–8] or a guttate psoriasis spared Becker's melanosis [9]. Also, a purpuric rash and a leukocytoclastic vasculitis that spared tattoos have been described [10, 11]. However, to the best of authors' knowledge, this is the first report of guttate psoriasis sparing tattoos. Remarkable is also the case of a patient with a reverse Koebner phenomenon, i.e., clearing of psoriasis lesions after tattooing [12]. All these observations, together with evidence for lower than anticipated KP risk in association with tattooing, indicate to a distinct pathophysiologic interaction of tattoo and psoriasis. We suggest that the macrophage, the key cell species involved in tattoo pathophysiology, is the cellular link of tattoo and the psoriasis sparing in this patient.

Dermal macrophages, both resident and blood-borne monocyte-derived, comprise a slowly renewing and in loco relatively long-lived cell species [13]. Macrophages are the key effector cells in the pathophysiology of tattoos, as they phagocytose and carry locally most of the tattoo pigment [14]. Moreover, they enable the stability of the tattoo via a process of capture-release-recapture of the pigment in situ during successive cell death and renewal cycles [15]. Zaba et al. [16] have previously shown that macrophages in normal skin that have ingested tattoo pigment was not able to stimulate T cell activation. This is probably the result of a LC3-associated phagocytosis (LAP) process [17], which ensures the shift of the local immune state towards sustained anti-inflammation by dampening of proinflammatory signals and preventing the presentation of autoantigens to other immune cells [18, 19]. Accordingly, a tattoo site can be conceptualized as a noninflammatory skin area with pigment-laden macrophages in a predominantly “deactivated,” immunologically inert, or “anti-inflammatory,” M2-polarized state [20], resembling a tissue milieu that corresponds to a sustained resolution phase of an inflammatory process.

On the other hand, macrophages emerge also as a key cell species in the pathophysiology of psoriasis. In human psoriasis, a subpopulation of classically activated proinflammatory macrophages plays a crucial role in the pathogenesis of skin lesions, and a preponderance of M1 macrophage activation state is also associated with increased PASI scores [21]. In accordance with above, markers of M2 macrophage polarization are reduced in psoriasis [22], and the improvement of psoriasis with TNF-*α* inhibitors correlates with the inhibition of the M1 macrophage polarization pathway [23]. Stimulated macrophages are the main immune-effector cell species in animal psoriasis models, like the inducible human TNF transgenic mouse line [24]. Furthermore, in the K14-VEGF-A-Tg mice psoriasis model, IL-35 therapy was shown to alleviate the development of skin lesions through the reduction of macrophage infiltration and a shift from a M1- to M2-dominated tissue milieu [25]. Finally, in the imiquimod psoriasis model, the skin lesions are characterized by a distinct accumulation of macrophages and monocytes with the concomitant activation of the proinflammatory M1 over the anti-inflammatory M2 macrophage polarization [26]. Within the framework of this latter model, the induction of a M2-polarization predominated tissue milieu may attenuate the development of psoriasis lesions [27].

Based on above evidence and recent findings, considering the relationship of autophagy and psoriasis lesions [22], we propose the hypothesis that tattoos represent a “psoriasis-hostile” tissue environment pertained by a population of LAP active M2-polarized macrophages. Expanding our above observation of guttate psoriasis tending to spar tattoo areas, we assume that further study of the relationship of psoriasis lesions to tattooed skin may provide important insights into the role of macrophages in the pathogenesis of this disease. However, sparing or not of a tattoo site by evolving psoriasis can be conclusively decided only during a generalized disease flare-up, like a guttate one, with lesions not just confided on the typical for psoriasis skin localizations, which generally do not comprise popular sites for tattooing. In the framework of the current world-wide “tattooing epidemic,” awareness is mandatory to document psoriasis cases in relation to tattooing and tattoos that may provide important insights into the role of the macrophage in the pathogenesis of psoriasis.

## Figures and Tables

**Table 1:** Effect of tattoo on psoriasis lesions centered Voronoi patches.

Skin site	CV^1^	Mean size (SD)^2^		*t*-test	Figure ^3^
		*V*_out_^4^	*V*_in_^5^	*P* value^6^	
Tattoo-free	0.46	13416 (6244)	N/A^7^	N/A	1
Tattoo #1	0.60	12878 (4791)	28972 (12205)	<0.001	2
Tattoo #2	1.23	15504 (8658)	6680000 (58326)	<0.001	3
Tattoo #3	0.73	12173 (9700)	25902 (14564)	<0.001	3

^1^CV, coefficient of variation of the sizes of the Voronoi patches. ^2^Mean size of Voronoi polygons (SD, standard deviation). ^3^Figure number in text, where the corresponding skin site is displayed. ^4^*V*_out_, Voronoi patches not crossing tattoo contours. ^5^*V*_in_, Voronoi patches that intersect with tattoo. ^6^*P* value for the comparison *V*_out_ vs. *V*_in_ (two-sample *t*-test). ^7^N/A, not applicable.
